# Medical knowledge, political tension, and social relevance: a content and framing analysis of vaccine-related TV broadcasts in the Philippines

**DOI:** 10.1136/bmjph-2024-002133

**Published:** 2025-07-25

**Authors:** Jonas Wachinger, Mark Donald C Reñosa, Georgina Janowski, Ma Leslie Ulmido, Jerric Rhazel Guevarra, Jeniffer Landicho, Shannon A McMahon

**Affiliations:** 1 Heidelberg Institute of Global Health, Heidelberg University Hospital, Heidelberg, Germany; 2 Department of Epidemiology and Biostatistics, Research Institute for Tropical Medicine, Muntinlupa City, Philippines; 3 Institute of International Health, Charité University Hospital, Berlin, Germany; 4 Department of International Health, Johns Hopkins University Bloomberg School of Public Health, Baltimore, Maryland, USA

**Keywords:** Vaccination, Communication, methods, Disease Outbreaks, Qualitative Research

## Abstract

**Introduction:**

Mass media plays a key role in shaping medical discourses on a societal level, and understanding this role could inform context-specific approaches to address current health challenges. However, public health scholarship that focuses on low- and middle-income countries, draws on media analytic approaches established in other fields, or utilises the potential of digital platforms for understanding non-print mass media remains limited. One context meriting a better understanding of media communication is vaccination in the Philippines, where large-scale discourses have repeatedly challenged vaccine confidence.

**Methods:**

To understand how vaccine information has been communicated on Philippine TV broadcasts, we systematically searched and extracted n=108 broadcasts using YouTube API queries. Our approach covered 16 years of broadcasting throughout three major waves of vaccination reporting including routine communication, the Dengvaxia controversy and its fallout, and the COVID-19 pandemic. We conducted a content analysis of the full dataset, followed by an in-depth qualitative framing analysis of 16 purposively selected broadcasts.

**Results:**

Our results highlight how broadcasts across periods of varying discourse intensity were generally pro-vaccine leaning. However, key vaccine information, such as regarding the safety and efficacy of immunisation, was often lacking. Framing of vaccination varied within and across broadcasts and over time as communication stakeholders (including broadcasters, medical or scientific professionals, political actors, and lay individuals) employed medical (eg, providing explanations of disease risks or vaccine functioning), political (eg, suggesting accountability and highlighting actions taken) and social frames (eg, emphasising belonging and community-level relevance).

**Conclusion:**

Vaccine messaging changes based on shifting societal discourses, stakeholders and communication objectives. Training health professionals to emphasise under-represented information and to purposively engage with prominent message frames could improve vaccination communication. Beyond vaccine hesitancy, our results also highlight how methodological advances can guide public health stakeholders analysing societal discourses and seeking to convey medical information to the broader population.

**WHAT IS ALREADY KNOWN ON THIS TOPIC**

- Existing public health scholarship emphasises the relevance of mass media communication for distributing health-related information and shaping societal discourses.
- To date, vaccination-related media analysis has predominantly focused on high-income country and/or print media; less is known about media messaging in low- and middle-income countries (many of which have recently experienced upheavals in vaccine confidence), and about broadcast media (which continues to be among the most-accessed types of media in many settings).
- Over the past two decades, vaccine confidence in the Philippines has been subject to several waves of contestation and recuperation co-shaped by large-scale media reporting, including during the Dengvaxia controversy and the COVID-19 pandemic.

**WHAT THIS STUDY ADDS**

- We provide detailed and context-specific insights into the content and framing of vaccine information on Philippine broadcast media over a timespan of 16 years.
- Broadcast content across phases is predominantly pro-vaccine leaning, but information is lacking regarding key vaccine characteristics relevant for decision-making (eg, regarding safety, efficacy and pricing).
- Communication stakeholders (broadcasters, medical professionals, political stakeholders and members of the civil society) employ medical, political and social frames to explain vaccines and diseases, to suggest accountability, or to emphasise belonging.

**HOW THIS STUDY MIGHT AFFECT RESEARCH, PRACTICE OR POLICY**

- Our results supplement the existing evidence to outline how media analysis in public health can provide novel and actionable insights into health-related mass media discourses.
- We provide methodological guidance on how to systematically search, extract and analyse health-related (broadcast) media using YouTube API queries and a combination of content and qualitative framing analysis.
- In light of burgeoning health mis- and disinformation we call on researchers and public health stakeholders to consider the content and framing of messaging in diverse contexts to develop novel communication approaches.

## Introduction

 The role of mass media in delivering health-related information is well documented, and targeted mass media campaigns have been shown to influence a spectrum of health behaviours in diverse populations.^1 2^ However, media communication also contributes to the co-construction of health and illness in a society more generally and can shape, reflect and even distort meaning-making processes within public discourses. ^3^ This meaning-making capacity of mass media has been highlighted particularly during public health emergencies, such as during the MERS-CoV or SARS-CoV-2 epidemics^4 5^ or in the context of highly contested health issues, such as family planning^6^ or vaccination.^7 8^

In the context of vaccination, concerns regarding growing vaccine hesitancy and its impact on public health efforts have increased globally in recent years,^9^,^11^ with several authors highlighting the prominent role of media-based vaccine (mis-) information.^12^,^15^ In this context, vaccine discourses in new mass media, including various social media platforms, have received considerable attention,^1316^,^19^ with a particular emphasis on the role of social media in spreading vaccine misinformation and challenging vaccine confidence, both during the COVID-19 pandemic^20^ and regarding other vaccination efforts.^21 22^

In comparison, information on vaccine narratives presented on traditional mass media platforms (eg, TV, newspapers, radio) remains limited, despite these platforms' continued role as sources for vaccine information in many settings,^23^,^25^ and of arguments emphasising traditional media's (at least historical) importance for filtering information for credibility and scientific accuracy.^21^ In light of this gap, a 2019 systematic review found that research analysing vaccine messaging in traditional mass media has been increasing, but commonly focuses on high-income countries, newspaper articles and the HPV vaccine.^26^ This current focus on high-income countries neglects the importance of examining setting-specific messaging in contexts facing large-scale vaccine controversies and service disruptions (including several low- and middle-income countries). Furthermore, given that large swaths of the global population continue to access traditional broadcast media (both offline and via online platforms), broadening the analytic scope beyond print media could provide valuable insights into prominent information sources relevant across societal strata.

Systematically understanding how vaccine information is presented on various media platforms could shed light on the dynamics underlying ongoing public immunisation discourses, but scholars have repeatedly emphasised that public health inquiry generally lacks utilisation of in-depth media analysis approaches already established in other fields.^3 27 28^ One such approach is framing analysis. Originating in cognitive psychology and anthropology, framing analysis proposes a toolbox to analyse how topics are promoted and discussed in different forms of media.^28 29^ In this context, ‘frame’ describes the specific way of presenting an issue, with an understanding that dominant frames over time can shape the societal understanding of said issue.^28^ As a result, frames form a key organising principle for an array of issue-related symbols,^30 31^ not only defining how important a given issue is considered to be, but also coining perceptions regarding its causes, consequences and solutions.^32^

While media framing analysis has since inspired scholarship in fields ranging from linguistics to management,^33 34^ its systematic application in public health contexts remains limited.^28^ In the context of vaccination specifically, notable examples of applying framing approaches include comparisons of lay and medical framings of immunisation in post-socialist Serbia,^35^ mainstream media communication on COVID-19 vaccines in Pakistan^36^ or newspapers’ framings of vaccination following novel legislation in Australia.^37^ In our study, we build on this work, contributing to filling the gaps in the literature related to framing analysis and traditional mass media analysis in public health and vaccination research, particularly in low-and middle-income countries. Based on a novel method for systematically searching in and extracting TV broadcasts from digital spaces and drawing on a combination of analytic approaches, we investigate vaccination messaging in Philippine TV network broadcasts over a span of 16 years.

## Methods

### Setting

In 2015, the Philippines was among the top ten countries worldwide for vaccine confidence.^11^ This trust in vaccines was challenged, however, in the context of the Dengvaxia controversy: in 2016, a novel Dengue vaccine was introduced in the country and subsequently administered to schoolchildren on a large scale. However, in November 2017, the vaccine’s developer Sanofi released new findings that the vaccine could increase the longer-term risk for severe Dengue infections among individuals without previous exposure to the virus, which in the high-endemic setting of the Philippines particularly included younger children.^38^,^40^ The release of this information led to a suspension of vaccination efforts and a large-scale vaccine scare, which was rapidly politicised and further fuelled by divisive or factually incorrect reporting in both social and traditional media.^41^ Following this scare, general vaccination rates in the country plummeted, facilitating subsequent outbreaks of measles and polio in 2018.^42 43^ While by 2020, vaccine confidence seemed to be rebounding,^11^ the onset of the COVID-19 pandemic, which in the Philippines was associated with a particularly long community lockdown and strict vaccine mandate, posed new challenges.^44 45^

Societal and health-related discourses feature prominently in Philippine mass media, particularly on TV, which remains the most used media platform in the country. In 2019, 96% of Filipinos ages 10–64 reported TV exposure, with a majority (66%) reporting daily exposure (social media was ranked second for exposure, with 74% of respondents reporting general exposure),^46^ and TV overall remains the most trusted type of mass media.^47^

### Media analysis

Our methodological approach entailed: (1) preparation, (2) data extraction and (3) data analysis.^48^ All data analysed for the purpose of this study are publicly available; no primary data were collected, thereby exempting this study from formal ethical approval requirements. An author reflexivity statement is included as online supplemental file 1.

#### Preparation

Based on a literature review on the role of TV broadcasts in the Filipino mediascape, and our own primary data collected on sources of vaccine information,^49^ we sought to examine the framing of vaccine information in public mass media and how frames developed over time and within and across highly publicised discourses. Acknowledging that vaccination attitudes are not exclusively shaped by targeted news or information formats, but also by the more cursory interactions with the topic in other formats, we further aimed to capture not only news reports, but a fuller spectrum of broadcasts including entertainment shows, telenovelas or talk shows.

#### Data extraction

Two TV conglomerates (ABS-CBN and GMA) capture a combined audience share of approximately 80% of the Philippine market.^50^ To the best of our knowledge, the websites of these dominant networks do not allow for systematically searching and extracting broadcasts; we therefore developed a Python script to extract broadcast data from YouTube, the online platform where networks consistently upload their broadcasts (for details of the Python code, see online supplemental file 2). As both networks have separate YouTube channels for different types of content (eg, focusing on entertainment, news, sports etc.), and as we wanted to cover content across these types, we selected a total of five channels for our study (detailed in online supplemental file 2) with a combined total of 122.8 million subscribers.

We aimed to cover broadcasts during and after highly controversial vaccine discourses, as well as amid phases of routine reporting when the topic was not of particularly high public interest. We therefore specified three adjoining timeframes: (1) the pre-Dengvaxia-controversy phase (after November 2006, when the earliest selected channel was created, and before 29 November 2017, when the Dengvaxia report was released), (2) the Dengvaxia and pre-COVID-19 phase (30 November 2017 to 30 January 2020, when COVID-19 was declared a public health emergency of international concern and the first case was confirmed in the Philippines) and (3) the COVID-19 phase (all videos uploaded after 31 January 2020 up to 1 August 2022 when data were extracted).

**Figure 1:**
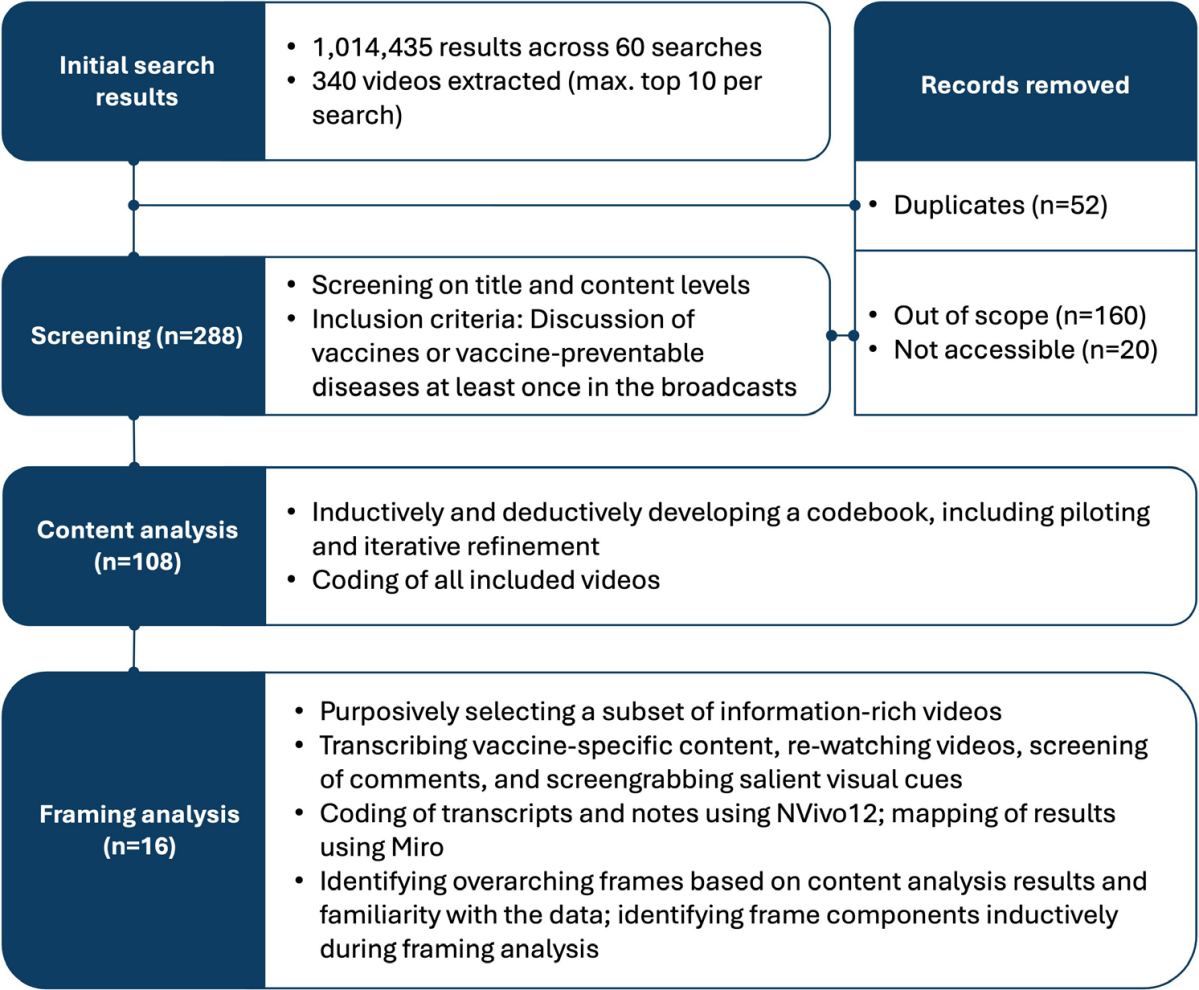
Flowchart of sampling and analysis process.

To develop the search string that underpinned the Python script, we worked with a biomedical librarian (JL) to identify key terms related to vaccination and vaccine-preventable diseases in both English and Filipino and piloted five search strings (see online supplemental file 2). Based on piloting results, the final search script extracted the top 10 results for each of the 60 possible combinations of the four search parameters (five channels, three timeframes, two search strings, two sorting approaches (by relevance and by view count)). All searches were conducted on 2 August 2022 (including videos uploaded until 1 August 2022) from a Filipino IP address using a newly created YouTube account and API key. Figure 1 presents a flowchart of how the final sample was identified and analysed. All stages were supplemented by reflexive notes, and co-authors in regular meetings validated decisions and discussed ambivalent cases until consensus was reached.

#### Data analysis

The final sample included for analysis consisted of n=108 videos (28, 40 and 40 videos from the three respective timeframes; see online supplemental file 3 for a full list of all selected videos). To analyse our dataset, we combined a content analysis of the full dataset with an in-depth qualitative framing analysis of a purposively selected subset of videos.^28^ For the content analysis, we developed a codebook based on inductively identified themes, as well as categories drawn from the existing literature on vaccine media messaging (eg, vaccine attitude, type of message, or risks and benefits discussed). Three Filipino-speaking team members applied the codebook prototype to five purposively selected videos, and results were discussed among co-authors to ensure a shared understanding of codes. Following codebook refinement, GJ, who is a native Filipino speaker, applied the codebook to the full dataset under the supervision of JW. Numerically coded data were analysed using Microsoft Excel 2019.

To identify a subset of videos for the subsequent framing analysis, team members performing the codebook piloting and first coding indicated particularly information-rich broadcasts to consider for more in-depth engagement. Of the n=36 identified broadcasts, JW and GJ then purposively selected broadcasts with the aim to represent the breadth of the data while also acknowledging proportionality of included broadcast types, contents and sentiments. The final subset of n=16 videos therefore included broadcasts from all three timeframes (2005–2017, n=4; 2017–2020, n=6; 2020–2022, n=6), different types of broadcasts (news reports, n=9; interviews/talkshows, n=3; entertainment formats, n=4) and broadcasts focusing on different vaccines and presenting different vaccine sentiments. We also included broadcasts with particularly high view, comment and like counts. Information power considerations^51^ helped us to strike a balance between dataset size and feasibility of in-depth qualitative engagement; online supplemental file 4 presents a full list of the videos selected for the framing component. JW and GJ rewatched selected videos, and GJ transcribed vaccine-specific parts verbatim and translated them into English. Transcripts included prominent audio-visual cues within vaccine-related broadcast sections. JW then analysed video transcripts following a framing approach, combining both inductive and deductive (based on content analysis results) facets.

Following the content analysis and the associated familiarisation with the full dataset, co-authors discussed core dynamics present in the data that could reflect overarching frames, including how vaccine information was not only presented as a medical topic, but also as relevant within societal and political spheres. Drawing on this understanding, and informed by framing research in other health-related contexts,^28 52^ we decided on three overarching frames (medical, social and political) to serve as analytic starting points. Building on these overarching frames, we then developed nuanced frame components^28^ via an in-depth engagement with the purposively selected videos, with the understanding that communicators could employ more than one frame within one broadcast.^28^ This approach allowed us to not only identify more nuanced dynamics of how frames interacted, but also to code for differences between individual speakers (eg, medical professionals vs lay people vs political stakeholders) within broadcasts. Coding of video transcripts and notes was done using NVivo 12, and results were mapped using a Miro-board (www.miro.com), supplemented by video screengrabs to integrate visual cues.

#### Patient and public involvement

Patients and the public were not directly involved in the study. However, the overarching aim of this research was to map vaccine communication in mass media, and the results presented thereby outline discourses in the public space.

## Results

### Video characteristics

Of the 108 videos analysed, all except three were predominantly in Filipino. The majority of videos were broadcasted on either ABS-CBN News (n=41) or GMA News (n=40), followed by GMA Public Affairs (n=17), GMA Network (n=5) and ABS-CBN Entertainment (n=4). View counts (range 361 to 2 237 043, median 9011), like counts (range 0 to 13 345, median 35.5) and comment counts (range 0 to 8681, median 10) generally increased across timeframes. On average, analysed videos lasted 4 min and 46 s (range 00:31 to 27:41 min), with the average duration of the individual video increasing from 3 min to 5 min and 52 s between the first and third timeframe. The earliest video included in the analysis was uploaded in July 2013, and the last was uploaded in July 2022.

### Content analysis

**Figure 2:**
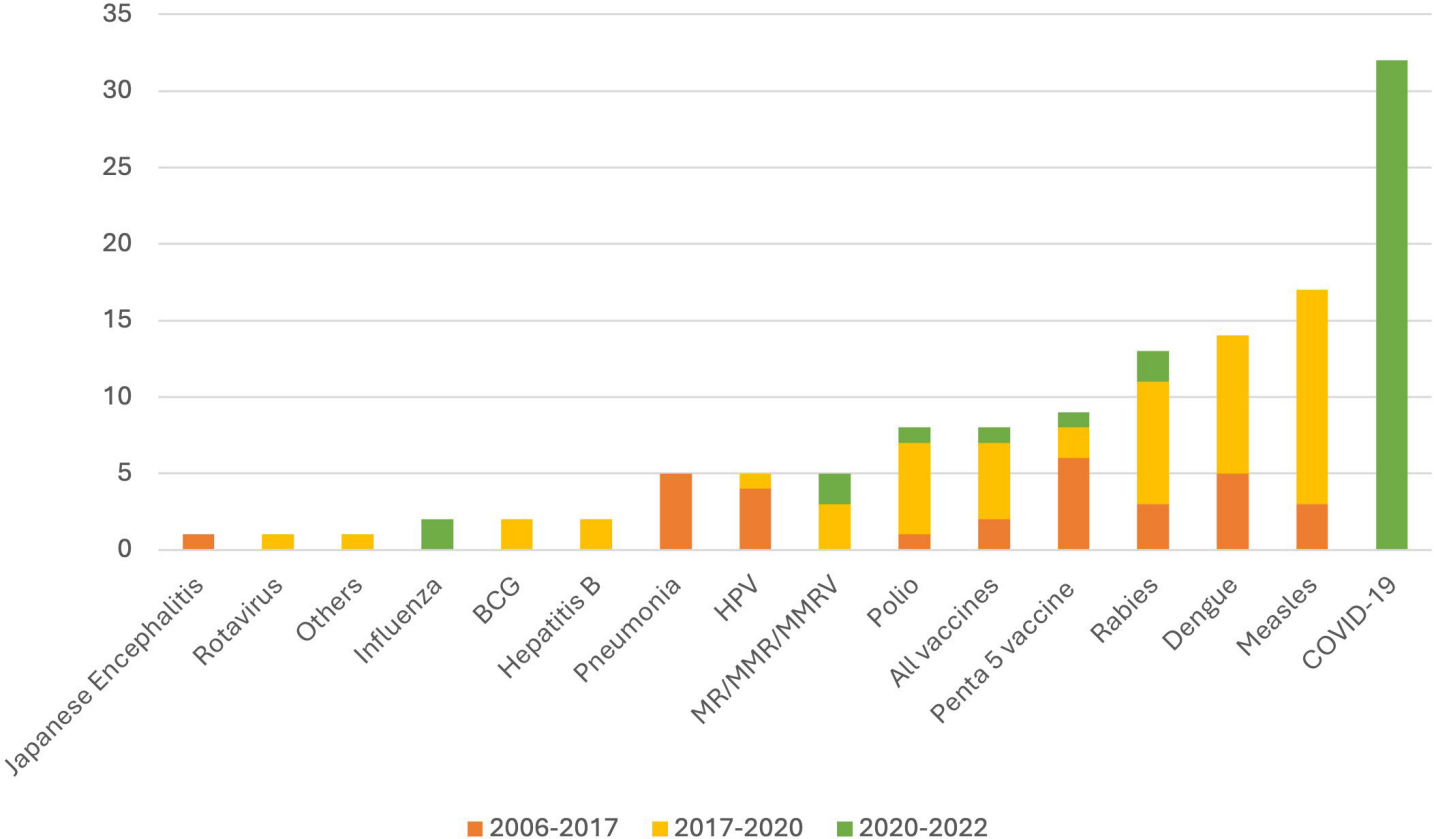
Vaccine-preventable diseases discussed across timeframes. Note: totals add to n=129, exceeding the number of analysed videos (n=108) as some broadcasts discussed more than one disease.

A majority of included videos presented generally positive vaccine attitudes (n=73). Half of the analysed videos (n=54) highlighted personal benefits of getting vaccinated (eg, protection against infection). n=27 videos mentioned a combination of personal benefits and benefits for others (eg, protection of close individuals or broader societal benefits), with the remaining n=27 videos not mentioning any vaccination benefits. Table 1 presents detailed results of our content analysis. Figure 2 highlights the vaccine-preventable diseases discussed across broadcasts and over time.

**Table 1:** Content analysis results

	2006–2017	2017–2020	2020–2022	Total
Vaccine attitude				
Clearly pro-vaccine	15	25	25	65
Leaning pro-vaccine	3	0	5	8
Neutral/no clear vaccine attitude/mixed	9	14	10	33
Leaning anti-vaccine	1	1	0	2
Clearly anti-vaccine	0	0	0	0
Main type of message				
News reports	23	33	34	90
Opinion broadcasts/interviews/talkshows	5	4	2	11
Entertainment shows	0	2	4	6
Documentaries	0	1	0	1
Vaccine effectiveness: explicit and (implied)*				
Vaccines are (mainly) effective	7 (14)	6 (14)	4 (10)	17 (38)
Vaccines are not effective	0 (0)	5 (6)	0 (0)	5 (6)
Mixed	0 (0)	1 (0)	3 (0)	4 (0)
Not explicitly commented on	21 (-)	28 (-)	33 (-)	82 (-)
Vaccine safety: explicit and (implied)*				
Vaccines are (mainly) safe	6 (17)	5 (13)	4 (9)	15 (39)
Vaccines are unsafe	0 (0)	5 (8)	1 (0)	6 (8)
Mixed	0 (0)	1 (0)	8 (0)	9 (0)
Not explicitly commented on	22 (-)	29 (-)	27 (-)	78 (-)
Benefits of vaccination				
Personal benefits (eg, protection from disease)	14	20	20	54
Combination of personal and other benefits (eg, protection of others, health system)	8	9	10	27
No benefits/not commented on	6	11	10	27
Risks of vaccination†				
None mentioned	15	25	25	65
Short-term side effects (eg, fever)	7	2	8	17
Profit motives, costs of vaccines	6	7	0	13
Death	1	6	2	9
Societal consequences (eg, increasing vaccine hesitancy)	0	8	0	8
Severe long-term side effects (eg, disability)	1	3	3	7
Ineffective or fake	0	5	1	6
Other, unspecified or unknown risks	0	0	4	4
Basis of authority and sources of broadcasted information†				
Scientists and scientific data	23	39	33	95
Medical professionals/doctors	18	32	29	79
Governmental or non-governmental organisations	21	31	24	76
Layperson/private experiences/personal stories	6	2	12	20
Legal expertise	2	4	1	7
Other	0	1	1	2

* For broadcasts that did not explicitly comment on vaccine effectiveness and safety, but which implicitly suggested a certain leaning, we included the implied effectiveness or safety notion in brackets.

† Broadcasts could mention more than one risk of vaccination or could invoke more than one basis of authority/source of information; totals in these sections therefore add up to more than n=108.

Overall, seven videos mentioned alternatives to vaccination. These alternatives commonly included general recommendations to ‘boost’ immune systems, for example by ensuring good nutrition, wearing protective clothes and insect repellant in the case of Dengue, and ensuring personal hygiene and ‘cleanliness’ of the living environment (including recommendations to avoid crowded places in case of highly infectious diseases such as measles). One broadcast, discussing supply shortages of human rabies vaccine, recommended to instead vaccinate pets with the readily available animal vaccine.

### Framing analysis

**Figure 3:**
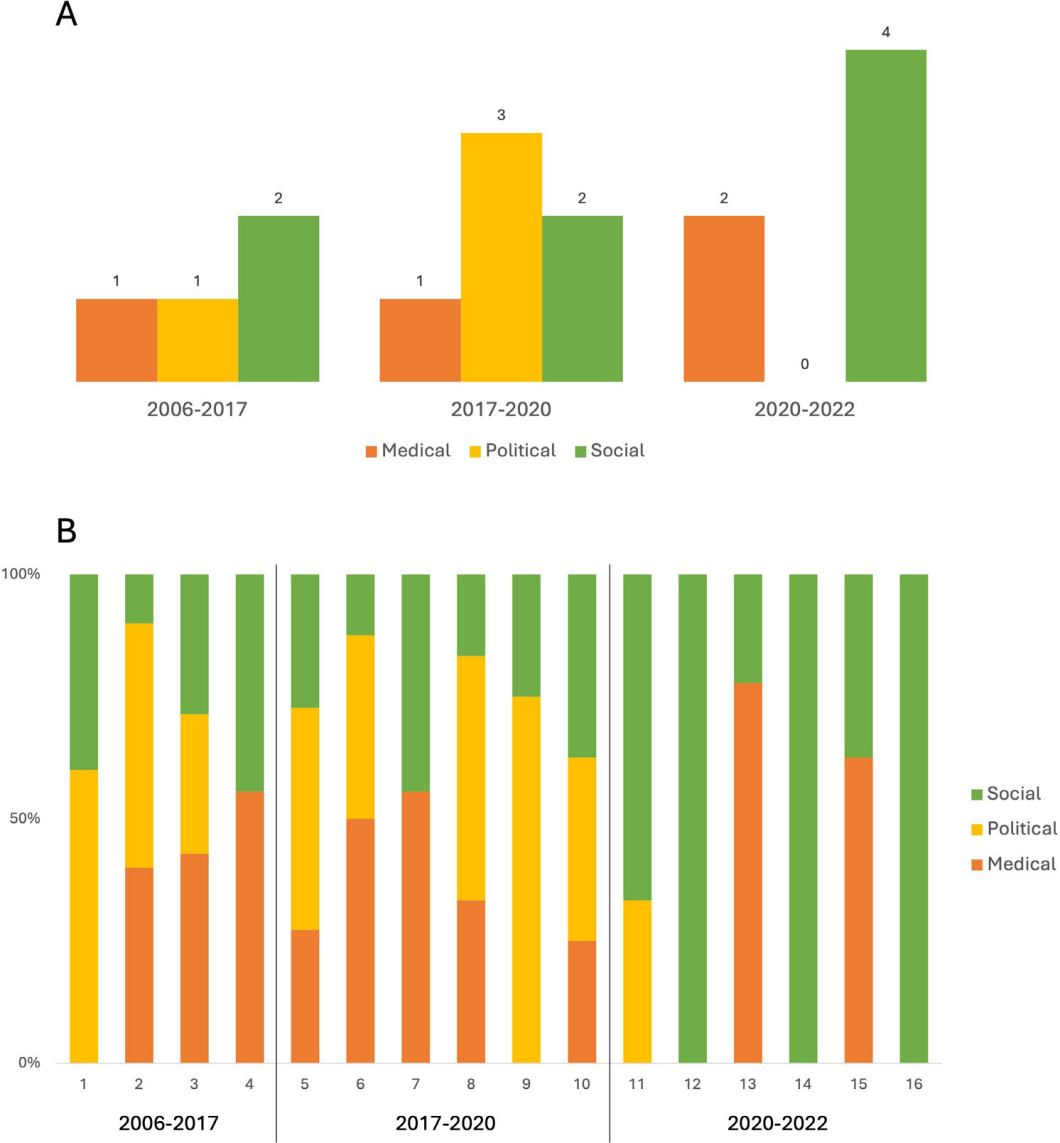
Frames employed in selected broadcasts over time. (**A**) Main frames employed in selected broadcasts. (**B**) Coexistence of frames in selected broadcasts, represented as a frame’s share (in %) of each broadcast’s coded components. Note: Timeframes include 2006–2017 (pre-Dengvaxia), 2017–2020 (Dengvaxia controversy) and 2020–2022 (COVID-19).

The n=16 selected broadcasts employed medical/scientific (eg, discussing medical risks and risk factors), political (eg, outlining decision-making processes and discussing accountability) and social (eg, highlighting social cohesion and collective responsibilities) frames when communicating vaccine information. When focusing on the main overarching frame being employed in a selected broadcast, the prominence of each of these frames varied across the analysed time periods (figure 3A).

Additionally, more than one frame or frame component was employed in almost all analysed broadcasts, often by various stakeholders. Figure 3B maps how framings coexisted in each individual broadcast (represented as a frame’s share of a broadcast's coded components included in the analytic mapping; broadcasts grouped by timeframes and sorted by upload date) to outline how different frames co-existed and competed for space in our sample. These maps highlight how, already prior to Dengvaxia, the political sphere of vaccination efforts competed with, and often overtook, medical information. This role of political framing further increased in the wake of the controversy, when stakeholders sought to establish political accountability for vaccine licensing and rollout. Exceptions to this inter-timeframe tendency included broadcasts exclusively focusing on providing information on one specific vaccine (eg, broadcasts focusing on vaccines against pneumonia (Video 4) or measles (Video 6; video numbers as per online supplemental file 4).

However, figure 3 also highlights how the employment of political frames sharply dropped in the context of COVID-19. At the same time, the social framing of vaccination saw an uptick during the COVID-19 pandemic, with an increasing number of broadcasts emphasising shared responsibilities and the relevance of vaccination for the broader community. This increase in social framings was also reflected in the emergence of new formats discussing vaccine-related topics: while earlier time periods were mainly coined by news reports and interviews, this repertoire now was supplemented by an increase in talk and entertainment shows. In several cases, these novel formats exclusively discussed the social dimension of vaccination (Videos 12, 14 and 16), excluding medical or political facets.

These dynamics across timeframes were also reflected in the prominence of, and information presented by, different stakeholders that were given voice in the selected broadcasts (besides the broadcasters themselves), including medical and scientific professionals, political stakeholders, and community members. Political stakeholders were particularly vocal during the Dengvaxia controversy, framing their information in both medical and political ways, while medical professionals largely refrained from entering the political sphere during and after Dengvaxia. In our sample, the engagement of political stakeholders dropped during the COVID-19 pandemic, associated with more prominence of both medical professionals and members of the general public.

On the more nuanced level of frame components, table 2 outlines how different stakeholders employed components throughout the individual broadcasts. In the remainder of this section, we present our findings along the columns of table 2. When referring to individual videos, we use video number (as per online supplemental file 4), upload date and broadcasting channel as identifiers.

**Table 2:** Framing analysis results

Nature of presenter	Frame 1. Medical and scientific	Frame 2. Political	Frame 3. Social
**Broadcasters**	**Delivering medical information** Symptoms, vectors, case counts, vaccination schedules **Highlighting importance of vaccination** for preventing diseases described as dangerous **Reporting medical and scientific developments** including clarifications of novel information and developments	**Contextualising political health targets and developments** including governmental health targets, schedules, concerns, and actions **Establishing accountability** and next investigative steps	**Emphasising belonging** by addressing viewers as ‘friends’ and sharing personal experiences **Highlighting the social dimension of vaccination** Emotionalised images, visualisation of hopes and challenges
**Medical professionals and scientists**	**Providing vaccination and disease information** including risks, transmission, treatment and associated concerns **Addressing concerns and communicating insecurities** by explaining scientific processes and showing vaccines and vaccination processes to address concerns,	**Criticising political processes** and voicing concerns regarding corruption and licensing approaches	**Emphasising belonging** by sharing stories as a fellow parent **Expressing respect, reliance, collaboration** Expressing thanks for getting vaccinated, outlining how achieving vaccination is a collaborative effort **Calling citizens to take action** by getting vaccinated and combatting vaccine hesitancy
**Political stakeholders**	**Communicating (medical) information** including via verbatim quotes from scientific studies **Shifting accountability and insecurity to medical sphere** ‘It is their fault’ and ‘there was no clear explanation’	**Explaining established processes** including pricing, licensing, taking (political) action **Outlining latest developments and justifying actions taken** ‘Will not stop’ to investigate, ‘on top of these issues’	**Emphasising belonging and placing citizens first** Prioritising informing fellow citizens over political day-to-day **Establishing social dimensions of vaccination and public responsibilities** Only so much ‘we‘ can do if people do not vaccinate and share misinformation
**Civil society and lay people**	**Employing medical frames to describe symptoms and risks** **Criticising medical processes** regarding vaccine licensing and injection	**Calling for and taking steps to ensure (political) consequences** Hotlines, recommendations, civil legal cases	**Explaining decision-making processes and own reasons to vaccinate** including via emotionalised images and stories **Highlighting vaccination fears** of children and parents

#### Medical and scientific frame

The medical and scientific frame was used by all involved stakeholders to present medical information about vaccination and vaccine-preventable diseases, including information on symptoms, vectors, case counts, risk factors and treatment options (see, eg, Video 13, 11.05.2021, ABS-CBN News, 1:34). Broadcasters and medical stakeholders often linked this information to direct calls for action by presenting recommended vaccination schedules or emphasising the topic as being ‘serious’ (Video 4, 25.11.2017, GMA Public Affairs) or ‘important’ (Video 5, 01.12.2017, ABS-CBN News): ‘Our point here is: The vaccine is so important and don’t wait until the COVID comes first’ (Video 15, 29.09.2021, GMA Network). The urgency of getting vaccinated was repeatedly emphasised via the medical frame, broadcasting images from symptoms or hospital wards (see, eg, Video 10, 01.03.2019, ABS-CBN News, 0:49).

Using the medical frame, stakeholders also clarified established processes (eg, testing protocols) for new vaccines. News broadcasters frequently reported on and clarified developments, for example, in the context of COVID-19 vaccine licensing or the emergence of the Dengvaxia controversy, and emphasised their efforts to deliver up-to-date and comprehensive information: ‘The GMA news still tries to ask Sanofi Pasteur for a statement’ (Video 6, 09.01.2018, GMA News). In comparison, medical stakeholders, while also discussing processes, often focused on alleviating medical concerns, for example by highlighting that ‘first of all, reactions are very common among vaccines’ (Video 13, 11.05.2021, ABS-CBN News) and directly showing vaccines to prove that they had not expired (see, eg, Video 3, 16.11.2017, GMA News, 1:21). Additionally, medical stakeholders communicated (scientific) insecurity rather openly as ‘for now we are still doing a study’ (Video 15, 29.09.2021, GMA Network).

Political stakeholders also discussed medical and scientific processes related to vaccination, but frequently employed the medical frame to shift accountability away from the political sphere: ‘The decisions that we need were actually based on the information, the documents and the data they shared with us’ (Video 2, 31.03.2016, ABS-CBN News). Especially in the context of the Dengvaxia controversy, political stakeholders highlighted that ‘there was no clear explanation’ (Video 5, 01.12.2017, ABS-CBN News) and that ‘it is not our fault. It is their fault’ (Video 8, 18.12.2018, ABS-CBN News). Building on this narrative, political stakeholders promised investigations to hold responsible parties accountable, ‘even if they are being pointed out by a group of doctors as the reason for the loss of people’s vaccine confidence’ (Video 8, 18.12.2018, ABS-CBN News). Similarly, lay people interviewed in the broadcasts employed medical frames when describing their symptoms and concerns, but also criticised medical licensing and vaccine administration processes, including concerns that vaccines are injected ‘with pressure’ (Video 3, 16.11.2017, GMA News).

#### Political frame

Broadcasters used the political frame to present specific vaccination targets (‘needed to be vaccinated are almost 2.5 million grade 1 students and 1.8 million grade 7 students’ (Video 1, 19.08.2015, ABS-CBN News)), as well as case counts and envisioned processes as communicated by government agencies: ‘DOH records in 2018 a drop of 44% from the total number of vaccinations in the Philippines. The lowest in the immunisation programme history of the agency, mainly due to Dengvaxia’ (Video 10, 01.03.2019, ABS-CBN News). Relying on direct quotes, interviews and statistics, broadcasters employed the political frame to outline actions taken, both by the government as well as by their channel, to present in-depth and critical reporting (see, eg, Video 5, 01.12.2017, ABS-CBN News, 0:22, and Video 6, 09.01.2018, GMA News, 1:59).

Political vaccine-related processes were, to a limited degree, also highlighted by medical stakeholders, for example in the context of critiques that political stakeholders would not heed, or wait for, scientific recommendations, including from international bodies such as the World Health Organization (Video 2, 31.03.2016, ABS-CBN News). Similarly, medical stakeholders voiced concerns regarding potential instances of corruption that might explain overpriced vaccine procurement. In comparison, political stakeholders often focused on explaining and justifying established processes: ‘Are we still going to wait for more children to die because of Dengue? Vaccines actually save lives. Why are we going to stop the government from preventing children from having Dengue, while in fact [the vaccine] is there?’ (Video 2, 31.03.2016, ABS-CBN News).

Discussions of accountability, especially during the Dengvaxia controversy, were a key politically framed topic across stakeholders. Broadcasters emphasised their efforts to present the ‘facts of the case’ (Video 5, 01.12.2017, ABS-CBN News), working with governmental agencies ‘for the truth to come out’ (Video 6, 09.01.2018, GMA News). Political actors themselves offered reassurances that ‘first of all’ they were ‘on top of these issues’ (Video 5, 01.12.2017, ABS-CBN News) and ‘will not stop to investigate’ (Video 8, 18.12.2018, ABS-CBN News), taking ‘all the possible means’ (Video 9, 12.02.2019, GMA News). Reassurances were often followed up with precise descriptions of the political steps taken, including the creation of task forces, working over weekends, halting vaccination rollout, or discussions with experts. Civil society members similarly called for concrete political actions as ‘we already have ideas who was responsible […] so in the civil case we will include those who are connected in allowing this’ (Video 6, 09.01.2018, GMA News), calling for individual-level consequences: ‘The [injecting] person’s license should be rebuked because of what they did to my child’ (Video 3, 16.11.2017, GMA News).

#### Social frame

Across stakeholders, a social frame was often employed to emphasise belonging to the general Filipino population. Broadcasters addressed their viewership as ‘dear friends’ and localised the vaccination topic (‘Let’s concern ourselves with the Philippines, ok?’ (Video 7, 09.12.2018, ABS-CBN News)). Similarly, several broadcast formats presented medical professionals as a fellow citizen sitting down to talk (see, eg, Video 4, 25.11.2017, GMA Public Affairs, 0:36), and doctors themselves addressed viewers as a fellow parent as ‘I had an experience when my son was 4 months old’ (Video 4, 25.11.2017, GMA Public Affairs). Political stakeholders emphasised how they would place the citizens first: ‘You know Ted, the health of the Dengvaxia vaccinated children covered by the Dengvaxia programme is the paramount of our consideration as of now’ (Video 5, 01.12.2017, ABS-CBN News).

In general, several broadcasts and stakeholders emphasised the social (and societally egalitarian) dimension of vaccines, particularly during the COVID-19 pandemic. A Christmas jingle featuring various celebrities promoted to ‘bakuna [engl: vaccinate] together’ (see, eg, Video 16, 24.11.2021, GMA Network, 0:25), and one host expressed the wish that ‘all Filipinos get vaccinated, right? Everyone…poor and rich. All, because no one is safe’, as then ‘our suffering will be over’ (Video 11, 18.12.2020 ABS-CBN Entertainment).

Presentation of images such as coffins and funerals of children whose deaths had been suspected to be linked to vaccination (see, eg, Video 3, 16.11.2017, GMA News, 0:43) further allowed for an emotionalised social framing of vaccines and was used to emphasise either risks or benefits of vaccination. Similarly, members of the general public explaining their decision-making processes often framed vaccination socially, highlighting how ‘my daughter needs me, so I took it for her’ (Video 13, 11.05.2021, ABS-CBN News), or how they vaccinated so that ‘many people would see that they shouldn’t be afraid to get vaccinated’ (Video 9, 12.02.2019, GMA News).

Medical professionals similarly employed the broadcast platform to directly call on citizens to act and to express gratitude and respect to those who had done so. At the same time, some medical professionals highlighted that ‘there’s [only] so much that we can do as doctors. We need help’ (Video 7, 09.12.2018, ABS-CBN News), as they had to rely on the openness and information shared by the parents when giving recommendations. In the same vein, political stakeholders argued that ‘people can’t be forced’ (Video 7, 09.12.2018, ABS-CBN News) and ‘to those spreading the news, the problem is not with us, the ones who will be affected by this are the children’ (Video 1, 19.08.2015, ABS-CBN News).

## Discussion

In our study, we systematically extracted vaccine-related Philippine TV broadcasts from YouTube and combined content and framing analytic approaches to analyse how vaccine information has been presented in Philippine TV over time. Our results highlight how vaccine information on TV is predominantly pro-vaccine leaning and presented in the form of news reports, and how highly publicised discourses (e.g. regarding specific vaccines such as the COVID-19 vaccine) can overshadow other vaccine-related reporting. Additionally, we outline how broadcasters, medical or scientific professionals, political stakeholders, and members of the general public employ medical (eg, for explaining diseases and vaccination principles or for emphasising urgency), political (eg, for establishing accountability and for declaring actions taken), and social (eg, to emotionalise reporting and to suggest belonging) framings of vaccine information.

Our results contribute to filling a gap in the existing literature on traditional mass media vaccine communication which has commonly focused on high-income countries and newspaper analysis.^26^ In the Philippine setting we are not aware of a systematic analysis of vaccine-related media discourses. Additionally, beyond one notable exception,^53^ available country-specific literature tends to focus on English-language newspapers.^54^ While we believe that such newspapers can reflect overarching discourses in the country, they might not represent the medium that Philippine citizens, particularly in rural areas, predominantly consult.^46 47^ Our work therefore contributes to the existing literature both on a national level, by providing vaccine-specific communication insights for content presented in the most-used and most-trusted mass media^46 47^ and, across settings, by bolstering our understanding of vaccine messaging beyond high-income and social or print media contexts.

Our manuscript also mirrors evidence available on vaccine-related TV coverage in high-income countries: a content analysis of H1N1-related TV coverage in Australia found a focus on conveying situation seriousness, governmental reassurances and concrete behavioural advice for viewers.^55^ Similarly, an investigation of nightly HPV vaccination TV coverage in the USA found broadcasts to frequently combine factual and personal or emotional information and outlined healthcare providers, government stakeholders and vaccine manufacturers to be key sources of information.^56^ Our results echo these findings by emphasising the prominence of medical and social frames in vaccine messaging, and regarding medical professionals, scientists and political actors being among the most vocal stakeholders in broadcasting media. However, while in other settings vaccine-related messaging in the media was highly politicised during the COVID-19 pandemic,^57 58^ our dataset instead highlighted a striking absence of predominantly politically framed broadcasts in the Philippines during this time. While this decrease in political frames may be associated with our methodological approach to search broadcast data using the YouTube algorithm, it may also be linked to setting-specific previous experiences with a heavily politicised vaccine debate and its fallouts during the Dengvaxia controversy.^41 59^ These experiences sparked calls for communication guidance and training for medical, government and media stakeholders,^41^ which to the best of our knowledge were at least partially implemented in the country prior to the COVID-19 vaccine rollout. These efforts might have contributed to a reduction in politicised reporting, and we encourage further exploration of the potential of targeted communication guidance based on setting-specific previous experiences to curtail political framings of medical information.

One study investigating vaccine information framing in the USA argued that parents’ vaccination attitudes are much more multidimensional than suggested by a continuum between vaccine rejecting and accepting.^60^ Our findings add to this argument by highlighting how medical, political and social frames do not necessarily align in the degree to which they promote vaccination but can co-exist in a single broadcast. We encourage future research to investigate the degree to which this coexistence of vaccine information frames could foster a further diversification in vaccine narratives. Our results additionally highlight how news reports dominate vaccine-related TV broadcasts in the Philippines, but also how the topic permeates other types of broadcasts including entertainment programmes, particularly with the onset of the COVID-19 pandemic. This mirrors previous research on the explicit or implicit integration of family planning information into entertainment broadcasts such as soap operas or telenovelas.^61^ Given the degree to which COVID-19 has impacted medial processes^62^ and how information overload might lead individuals to avoid news to preserve mental well-being in times of crisis,^63^ we encourage further research on how health-related topics in general and vaccination in particular are featured in pop culture and entertainment media. Such research would not only further our understanding of implicit meaning-making but also develop novel pathways towards leveraging the potential of ‘edutainment’ for scientific communication.^61^

In general, a majority of analysed broadcasts in our study were pro-vaccine leaning, with many mixed and relatively few clear anti-vaccine broadcasts; these findings align with previous research related to English-language HPV vaccine broadcast media content on YouTube, which tended to be more positive than user-generated content.^17^ At the same time, research on general YouTube content regarding the HPV vaccine^64^ or vaccination more broadly^65^ found large proportions of videos to be negatively connotated. Our results therefore provide additional insights into the role of broadcast media as content contributors in an online sphere: While a considerable body of literature has engaged with vaccine-related YouTube content^1718 64^,^66^ and health-related YouTube videos in general,^67^ we are not aware of other studies focusing exclusively on broadcast media YouTube content to assess setting-specific TV vaccine coverage. We encourage future research that explores differences in online health messaging (particularly between content uploaded by traditional mass media channels and by independent users) and between traditional mass media content presented in linear broadcasts and in online spaces.

Beyond the specific use case of analysing vaccine-related broadcast data, our methodological approach also emphasises the potential of online and open-access datasets to bolster traditional mass media analysis. While a subset of mass media data is archived in readily searchable repositories, such repositories in our experience predominantly focus on print and/or English-language media, often combined with high access fees. Particularly for research on media communication in diverse settings, systematically searching and extracting data available on open-access platforms (such as YouTube for broadcast media in our case) could therefore supplement working with large-scale efforts for media archiving and listening, such as the GDELT project.^68^ At the same time, the ‘data gold rush’^69^ sparked by the broad accessibility of online datasets can also present challenges for the systematic extraction and analysis of datasets, particularly in the context of in-depth but work-intensive qualitative approaches. Our approach of combining multiple steps of data extraction and analysis could therefore provide new avenues for making large datasets accessible for in-depth qualitative research; we encourage researchers to build on our experiences, both by employing our approach to combining content and framing analysis to other research questions and media types, and by expanding this approach with other analytic pathways for understanding media reach, content and impact.

### Limitations

This study has limitations. First, our search approach employs YouTube channel uploads as a proxy for content broadcasted in Philippine TV. While this allowed us to search and analyse media broadcasted in the country in, to the best of our knowledge, an unprecedentedly systematic way, it might also have led to the exclusion of broadcast not uploaded to or since removed from YouTube. Our newly developed search approach also required us to rely on the not-always-exhaustive available information regarding the functioning of the YouTube API and algorithms. Scholars have highlighted how social media platforms have undertaken some efforts for clearer monitoring of available content and how this might not only change the content accessed by users, but in turn also might restrict datasets available to researchers.^48^ While the opacity of YouTube algorithms might have been less problematic in the case of our study, as we focused on all content uploaded by preselected specific channels, we cannot exclude the possibility for certain platform-specific functions to have impacted our search results.

Second, in our analysis, we focused on national-level TV channels with a routinely updated YouTube presence that were affiliated with one of the two major Philippine broadcasting conglomerates. While these conglomerates together account for the vast majority of TV market share in the Philippines, and the combined subscriptions to their YouTube channels exceed the country’s population, we acknowledge the possibility that this choice of data source might have introduced biases. We therefore encourage further broadcast media analyses that focus on smaller TV channels of high relevance in specific regions or on broadcasting data sources beyond YouTube.

Third, as is often the case with seemingly infinite media datasets extracted online, the work-intensive nature of in-depth qualitative framing analysis required us to focus on a data subset. However, we tried to mitigate this challenge via our approach of combining the systematic search and extraction of relevant datapoints with a preliminary content analysis of the full dataset and a purposive selection of datapoints for the subsequent in-depth framing analysis. Future large-scale quantitative efforts (eg, also drawing from additional data sources or media types) could supplement our qualitative work. Finally, the Philippine setting combines several unique characteristics regarding media landscape and previous vaccine discourses; generalisations of our results to other settings or health-related issues therefore might merit caution.

## Conclusion

In this study, we analysed how vaccine information was presented in Philippine broadcast media across periods of routine messaging, highly politicised vaccine discourses, and global public health emergencies. Our findings emphasise how, despite a generally positive tenor of TV vaccine-reporting, a range of stakeholders employ medical, social and political frames to shape message emphasis. In light of mass media’s prominent role in public health discourses, we encourage future research to build on our findings, including by testing the impact of specific frames on vaccine attitudes and uptake, by exploring the dynamics of power underlying frame setting, or by employing our approach (to combine content and framing analysis to engage with mass-media datasets extracted from openly available online repositories via API-based search queries) to analyse media content in other geographical settings or health contexts. In the wake of the COVID-19 pandemic and the reappraisal of public health communication more broadly, we also call on public health stakeholders and policymakers to consider the role of media-based message framing, including for the purpose of targeted communication trainings for health practitioners or for developing approaches to challenge specific frames relevant to their respective contexts. With our work, we aim to encourage comprehensive media analyses in public health scholarship, thereby facilitating stakeholders’ ability to acknowledge and rapidly respond to health-related discussions underway in the public space.

## Supplementary material

10.1136/bmjph-2024-002133 online supplemental file 1

10.1136/bmjph-2024-002133 online supplemental file 2

10.1136/bmjph-2024-002133 online supplemental file 3

10.1136/bmjph-2024-002133 online supplemental file 4

## Data Availability

All data relevant to the study are included in the article or uploaded as supplementary information.
