# Supplementary material for: Medical knowledge, political tension, and social relevance: a content and framing analysis of vaccine-related TV broadcasts in the Philippines
Source: BMJ Public Health. 2025 Jul 25;3(2):e002133. doi: 10.1136/bmjph-2024-002133 (PMC12306340; doi:10.1136/bmjph-2024-002133)
Supplement: online supplemental file 1 [file bmjph-3-2-s001.pdf]

Medical knowledge, political tension, and social relevance: a content and framing analysis of vaccine-related TV broadcasts in the Philippines.

Supplemental file 1. Author reflexivity statement.

1. **How does this study address local research and policy priorities?**

This study was designed by researchers from the Heidelberg Institute of Global Health (HIGH), Heidelberg, Germany, and the Research Institute for Tropical Medicine (RITM), Muntinlupa, the Philippines. Vaccine hesitancy is one research and policy focus identified by RITM (the research arm of the Philippines Department of Health (DOH)) following the Dengvaxia controversy.

2. **How were local researchers involved in study design?**

The Dengvaxia controversy and its fallout became a major topic in the Philippines, in courses related to global health, and in regular conversations between MDR, SAM and the broader team. These conversations sparked the broader project SALUBONG, focusing on the human-centered design of a vaccine confidence intervention. Over the course of this project, vaccine communication in Philippine mass media repeatedly emerged as a source of information underrepresented in the academic literature. This insight sparked the design of the present study, which was coined by iterative exchanges between all members of the study team from both LMICs and HICs. Local researchers therefore contributed to all aspects of the study design, including the identification of prominent news platforms, search strings, technical extraction approaches, and data analysis and interpretation.

3. **How has funding been used to support the local research team?**

This specific research component did not receive external funding beyond JW's personal doctoral scholarship; one local researcher (GJ) was supported using internal funds. However, the larger research project underlying the present work generated approximately 100,000 USD (via a grant from the Gates Foundation). All decisions on funding allocation were made in partnership between HIC and LMIC research partners; a majority of funds were used for data collection and local design of intervention material.

4. **How are research staff who conducted data collection acknowledged?**

All researchers who were involved in the extraction and analysis of data and meet the ICMJE criteria have co-authored this manuscript. Two colleagues (one each based in the Philippines and in Germany) with contributions not meeting ICMJE co-authorship criteria are acknowledged in the acknowledgment section.

5. **Do all members of the research partnership have access to study data?**

All analyzed data are publicly available; all members of the partnership therefore have access to the full dataset.

6. **How was data used to develop analytical skills within the partnership?**

Local partners were involved in all steps of the data analysis process. This work was marked by mutual learning (between HIC and LMIC colleagues) and the co-development of a novel

data extraction and analytic approach. Additionally, the overarching partnership between HIGH and RITM is ongoing, with several capacity building activities for quantitative and qualitative analysis as well as academic writing already completed or planned for the future.

7. How have research partners collaborated in interpreting study data?

All research partners contributed to the interpretation of the data. Building on the newly developed data extraction and analysis approach, the identification, interpretation, and presentation of media frames of vaccine information were iteratively discussed within the broader research team. Co-authors also provided critical feedback on the manuscript across several versions to ensure contextualized interpretation of study data.

8. How were research partners supported to develop writing skills?

Throughout the overarching collaboration underpinning this article (see points 2 and 3), SAM, who has additional training as a journalist, provided scientific writing guidance to all team members. Several local early career research team members, 3 of whom are co-authors on this article, have already published first-authored publications on data originating from the larger collaboration.

9. How will research products be shared to address local needs?

This article will be published open access. Additionally, results from the larger collaboration are routinely shared in the form of policy briefs with the local policymaking bodies. Finally, we have repeatedly presented our findings to RITM and DOH in several forums, including at academic conferences and in bilateral meetings.

10. How is the leadership, contribution and ownership of this work by LMIC researchers recognised within the authorship?

Besides JW and SAM, all co-authors of this article are LMIC researchers. However, as this article originates from JW's doctoral studies and is part of his dissertation supervised by SAM, JW and SAM are first and last authors of this publication. Local researchers involved in the data collection, analysis, and/or writeup have co-authored this manuscript (see point 4).

11. How have early career researchers across the partnership been included within the authorship team?

All co-authors of this manuscript except SAM are early career researchers (Defined as having less than 5 years of experience following completion of postgraduate research training). JW and MDR have recently completed their doctoral research. All other co-authors are in the pre-doctoral phases of their careers.

12. How has gender balance been addressed within the authorship?

The authors of this manuscript identify as non-binary (1), male (2), and female (4). We acknowledge that, with most co-authors identifying as female, a skewness in gender representation exists in the authorship team; however, given the historically heavily patriarchal structures in the academic system, we do not see this as an inherently problematic imbalance. Nevertheless, throughout the writing and publication processes, we aimed to facilitate open conversation and reflection to facilitate a balanced representation of perspectives across the spectrum represented in the authorship team.

13. How has the project contributed to training of LMIC researchers?

As highlighted in point 11, all authors besides SAM are early career researchers. Two LMIC co-authors (GJ and MLU) contributed to this work while pursuing their Master's in International Health, and received in-depth training on the analytic approaches informing this manuscript as part of their work. Furthermore, as part of the broader partnership between HIGH and RITM, several training activities for LMIC researchers have taken place both in the Philippines and in Germany.

14. How has the project contributed to improvements in local infrastructure?

This project has not directly contributed to improvements in local infrastructure.

15. What safeguarding procedures were used to protect local study participants and researchers?

No primary data was collected for this research, and no identifiable information of any individual YouTube user was extracted. Routine meetings of the broader research team were held to identify and address potentially emerging risks; we are not aware of any additional risks individuals or team members incurred based on this work.
