# Supplementary material for: Medical knowledge, political tension, and social relevance: a content and framing analysis of vaccine-related TV broadcasts in the Philippines
Source: BMJ Public Health. 2025 Jul 25;3(2):e002133. doi: 10.1136/bmjph-2024-002133 (PMC12306340; doi:10.1136/bmjph-2024-002133)
Supplement: online supplemental file 2 [file bmjph-3-2-s002.pdf]

Medical knowledge, political tension, and social relevance: a content and framing analysis of vaccine-related TV broadcasts in the Philippines.

Supplemental file 2. Search approach development.

1. Channel selection

We selected five main channels belonging to the two TV networks, including:

- ABS-CBN News (@abscbnnews, created October 22, 2009, 14.9 million subscribers),
- ABS-CBN Entertainment (@abscbnentertainment, created July 16, 2008, 43.3 million subscribers),
- GMA Network (@gmanetwork, created November 20, 2006, 30.7 million subscribers),
- GMA Integrated News (@gmanews, created October 29, 2007, 13.1 million subscribers), and
- GMA Public Affairs (@gmapublicaffairs, created August 8, 2009, 20.8 million subscribers).

We excluded channels associated with the two networks that had purely regional focus (e.g., GMA Regional TV), were focused on sports broadcasts, music, or cinema (e.g. ABS-CBN sports, ABS-CBN Star Music), or had less than one million subscribers at the time of data extraction (e.g. ABS-CBN Talk or ABS-CBN Lifestyle).

2. Python script development

To systematically search and extract vaccine-related broadcasts during the specified timeframes from the selected channels, we developed a Python script which utilizes the YouTube Application Programming Interface (API). The script allows for defining search parameters in terms of date and channel of video upload, as well as search terms (combined, according to YouTube API documentation, by Boolean operators OR or NOT). Additionally, the script allows for specifying whether the videos should be sorted by the number of views, or by the relevance for the search terms according to YouTube's algorithm, and extracts information regarding video view-, like-, and comment-counts.

3. **Search string piloting**

We piloted five search strings to account for potential differences between YouTube search algorithms and established search approaches for scientific literature (see Table S1). While the shortest search string performed best in terms of identifying the highest absolute number or relevant videos, the longest search string yielded the least results but the highest proportion of relevant results. We therefore decided to include both search strings and to remove potential duplicate results in a second step.

Table S1: Piloted search strings

	Search string	Characteristics	Total search results	Extracted videos	Relevant videos
1	Vaccine *OR* bakuna	Including only the term 'vaccine' in both English and Filipino	466,298	50	32 (64%)
2	Vaccination *OR* vaccine *OR* vaccines *OR* immunization *OR* immunisation *OR* bakuna *OR* pagbabakuna *OR* turok	Including vaccine-related terms in both English and Filipino	727,398	50	22 (44%)
3	Vaccination *OR* vaccine *OR* vaccines *OR* immunization *OR* immunisation *OR* bakuna *OR* pagbabakuna *OR* turok *OR* Polio *OR* Measles *OR* Tetanus *OR* Mumps *OR* Rubella *OR* Tuberculosis *OR* Hepatitis B *OR* Diphtheria *OR* Pertussis *OR* Meningitis *OR* Pneumonia *OR* Influenza B	Including vaccine-related terms in both English and Filipino, as well as the names of vaccine-preventable diseases covered by routine childhood immunization in the country	439,135	50	14 (28%)
4	Vaccination *OR* vaccine *OR* vaccines *OR* immunization *OR* immunisation *OR* bakuna *OR* pagbabakuna *OR* turok *OR* Polio vaccine *OR* "bakuna sa polio" *OR* Measles vaccine *OR* "bakuna sa tigdas" *OR* Tetanus vaccine *OR* bakuna sa tetano *OR* Mumps vaccine *OR* Rubella vaccine *OR* MMR vaccine *OR* Tuberculosis vaccine *OR* BCG vaccine *OR* "bakuna sa tuberculosis" *OR* Hepatitis B vaccine *OR* Diphtheria vaccine *OR* Pertussis vaccine *OR* Meningitis vaccine *OR* Pneumonia vaccine *OR* Influenza B vaccine	Developed following best-practice approaches for scientific systematic literature searches to identify videos addressing the topic of vaccination and vaccine-preventable childhood illnesses.	73	28	21 (75%)

Note: Search string 4 extracted less than 50 videos despite the total results being >50 as some individual channels yielded less than 10 results.
