# Supplementary material for: Medical knowledge, political tension, and social relevance: a content and framing analysis of vaccine-related TV broadcasts in the Philippines
Source: BMJ Public Health. 2025 Jul 25;3(2):e002133. doi: 10.1136/bmjph-2024-002133 (PMC12306340; doi:10.1136/bmjph-2024-002133)
Supplement: online supplemental file 3 [file bmjph-3-2-s003.pdf]

Medical knowledge, political tension, and social relevance: a content and framing analysis of vaccine-related TV broadcasts in the Philippines.

Supplemental file 3. Final set of extracted videos for content analysis.

Table S2: Final set of extracted videos for content analysis

	Name	Upload Date	Channel	Views	Comments
1	Bandila: Senior citizens get free vaccine vs pneumonia<br>https://www.youtube.com/watch?v=vIgyplioCHY	27.05.16	ABS-CBN News	8321	0
2	TV Patrol: DOH, may libreng bakuna kontra HPV<br>https://www.youtube.com/watch?v=mkNq68iCgpw	25.05.16	ABS-CBN News	15205	7
3	Bakuna kontra HPV sa mga paaralan, ibabalik ng DOH<br>https://www.youtube.com/watch?v=U1Ct_Xp3DZY	18.05.17	ABS-CBN News	3437	2
4	TV Patrol: Anti-dengue vaccine, ligtas ba?<br>https://www.youtube.com/watch?v=rYWfhZ3HbXg	31.03.16	ABS-CBN News	9919	11
5	Penta-5 vaccine, kulang ng supply?<br>https://www.youtube.com/watch?v=NQUY--QO6IU	23.08.15	ABS-CBN News	21118	0
6	DOH: Ligtas ang libreng bakuna<br>https://www.youtube.com/watch?v=ZFlSVTO3FO0	19.08.15	ABS-CBN News	8985	0
7	Bandila: DOH denies dengue vaccine caused baby's death<br>https://www.youtube.com/watch?v=JOCEs_BVzTk	26.04.16	ABS-CBN News	5343	3
8	Bandila: Pinoys to get anti-dengue vaccine<br>https://www.youtube.com/watch?v=v-OAUcKrqyI	05.01.16	ABS-CBN News	1356	0
9	Bandila: Expert raises concern over safety of dengue vaccine<br>https://www.youtube.com/watch?v=viLv_aTa2U0	31.03.16	ABS-CBN News	1907	2
10	Poor Señorita: Rabies vaccine<br>https://www.youtube.com/watch?v=Qqmv2wtbsT0	07.04.16	GMANetwork	31961	2
11	UB: Dr. Tayag: May bakuna sa diphtheria<br>https://www.youtube.com/watch?v=50yimU23FoQ	03.02.17	GMA News	2418	3
12	Pagtaas ng kaso ng tigdas, inaalam kung may kinalaman sa pagdagsa ng Yolanda victims sa Kamaynilaan<br>https://www.youtube.com/watch?v=Cuz1AXOVph4	19.12.13	GMA News	953	0
13	Lapu-Lapu City LGU, ipinatigil muna ang pamimigay ng bakuna sa health center<br>https://www.youtube.com/watch?v=d1RA-kEvahc	16.11.17	GMA News	541	1
14	BT: Pagkamatay ng 2 sanggol dahil umano sa bakuna, iniimbestigahan ng DOH<br>https://www.youtube.com/watch?v=1lk9U33NDzI	16.11.17	GMA News	33862	24
15	KB: Anti-Pneumonia vaccine para sa mga sanggol, libre nang ibibigay ng DOH<br>https://www.youtube.com/watch?v=8Egprqz9GYg	18.07.13	GMA News	591	0
16	Saksi: Sec. Ona: mas mura noong 2012 biniling pneumonia vaccine ng DOH<br>https://www.youtube.com/watch?v=5VxxCNDhhek	11.11.14	GMA News	1025	0

17	UB: Bakuna vs. tigdas at polio, libre sa mga brgy. health center https://www.youtube.com/watch?v=lP_RG2lVRhQ	04.09.14	GMA News	15304	2
18	BT: Mga kagat ng hayop, hindi dapat ipagwalang bahala kahit may bakuna ang nakakagat https://www.youtube.com/watch?v=tqwMchj90Lc	04.09.14	GMA News	3009	0
19	Saksi: Pangamba ng ilang doktor, hindi ligtas ang dengue vaccine na libreng ibinigay ng DOH https://www.youtube.com/watch?v=3Lj_o52hKws	15.11.16	GMA News	6081	16
20	SONA: Pagbili ng DOH ng mga bakuna sa pneumonia, pinapaimbestigahan ni PNoy https://www.youtube.com/watch?v=8bxkDZCzKj4	10.11.14	GMA News	512	1
21	BP: Libreng bakuna para sa mga aso, ibinigay ng lokal na pamahalaan https://www.youtube.com/watch?v=q5BZ4jf8iYo	14.03.14	GMA News	1380	0
22	24 Oras: DOH, nagbabala laban sa pekeng bakuna sa tigdas https://www.youtube.com/watch?v=bUdxj0XE6E0	18.01.14	GMA News	1692	0
23	Unang Hirit: Talakayan kasama si Dr. Eric Tayag ukol sa Diphtheria https://www.youtube.com/watch?v=pnfHBhBIJhU	03.02.17	GMA Public Affairs	11879	8
24	Pinoy MD: Mga sintomas at paraan para maiwasan ang cervical cancer https://www.youtube.com/watch?v=9TjUPlVcb1c	14.11.16	GMA Public Affairs	758796	237
25	Unang Hirit: Panganib dulot ng tetano https://www.youtube.com/watch?v=Y-WFXxzQhs4	12.11.13	GMA Public Affairs	73464	34
26	Pinoy MD: Sakit na pneumonia, paano nga ba maiiwasan? https://www.youtube.com/watch?v=RfU7wGtNvZY	25.11.17	GMA Public Affairs	461221	109
27	Unang Hirit: Kapuso Kalusugan: Paano maiiwasan ang Japanese Encephalitis? https://www.youtube.com/watch?v=Pw0v44rTqoI	05.09.17	GMA Public Affairs	6219	0
28	Pinoy MD: How to detect cervical cancer? https://www.youtube.com/watch?v=F5tas6aePHA	20.07.16	GMA Public Affairs	60049	32
29	Salamat Dok: Importance of measles vaccines https://www.youtube.com/watch?v=5HZ4Ny2c1LM	09.12.18	ABS-CBN News	10089	3
30	Salamat Dok: Dr. Monteiro highlights the importance of vaccination against measles https://www.youtube.com/watch?v=dw63NrydjaA	24.02.19	ABS-CBN News	5647	2
31	Early Edition: Adult patient dies due to measles complications - health expert https://www.youtube.com/watch?v=lZ26vc75BeU	11.02.19	ABS-CBN News	1635	4
32	Bandila: DOH - Kumpletuhin ang bakuna ng mga bata https://www.youtube.com/watch?v=L6NdJSGD13k	01.03.19	ABS-CBN News	8918	5
33	Failon Ngayon: Anti-rabies Vaccines https://www.youtube.com/watch?v=isRNVpTRI08	21.04.18	ABS-CBN News	82272	56
34	Philippines suspends dengue vaccine program after Sanofi warns of risks https://www.youtube.com/watch?v=n5qoqKZNvGM	01.12.17	ABS-CBN News	22231	49
35	TV Patrol: Allergic reaction ng aso sa bakuna inireklamo https://www.youtube.com/watch?v=nzeQMJNbVSY	04.02.19	ABS-CBN News	3995	7
36	TV Patrol: Bakuna kontra cervical cancer, di dapat katakutan – DOH https://www.youtube.com/watch?v=uLlGWdoL3Ts	12.11.18	ABS-CBN News	2755	21
37	TV Patrol: Bakuna kontra dengue, maaaring makasama sa ilan: pag-aaral https://www.youtube.com/watch?v=Mwj1wRG1rjA	30.11.17	ABS-CBN News	6259	9

38	TV Patrol: Ilang magulang, tumanggi sa bakuna vs tigdas para sa anak<br>https://www.youtube.com/watch?v=qhf-avZicIA	25.04.18	ABS-CBN News	3212	4
39	DOH nagbabala sa pagbabalik ng polio dahil sa takot sa bakuna | TV Patrol<br>https://www.youtube.com/watch?v=t3Uk8X-yMls	31.08.19	ABS-CBN News	5518	5
40	Dr. Marizel Wong talks about the importance of polio vaccine | Salamat Dok<br>https://www.youtube.com/watch?v=zU50RgAh2vM	20.10.19	ABS-CBN News	1775	2
41	TV Patrol: PAO itinangging sila ang dahilan ng 'vaccine scare'<br>https://www.youtube.com/watch?v=e6HTw-ERTsY	18.12.18	ABS-CBN News	822	3
42	Bubble Gang: Sigang takot sa injection<br>https://www.youtube.com/watch?v=wC_MJ4Lacsw	16.02.18	GMANetwork	850922	165
43	The Cure: Full Episode 46<br>https://www.youtube.com/watch?v=XSo9wXxR6y4	29.06.19	GMANetwork	94769	62
44	Dr. Rey Salinel lists down the symptoms of measles and dengue | Magandang Buhay<br>https://www.youtube.com/watch?v=iTjC8W8DTaM	01.03.19	ABS-CBN Entertainment	99837	30
45	24 Oras: Internal organs ng batang namatay umano, nadiskubreng hindi naibalik ng ospital<br>https://www.youtube.com/watch?v=qbIvl8SA6c8	03.01.20	GMA News	1611486	1836
46	Batang nasawi matapos maturukan ng Dengvaxia, nakitaan din ng labis na pagdurugo sa katawan<br>https://www.youtube.com/watch?v=zh8p_eSHHPM	11.01.18	GMA News	865197	323
47	Matatandang nagkaka-tigdas sa San Lazaro Hospital, dumami<br>https://www.youtube.com/watch?v=K6FARmmrCOA	19.02.19	GMA News	5677	2
48	Pati mga fast food chain, nilagyan na ng vaccination site<br>https://www.youtube.com/watch?v=nu9rgVWopLM	12.02.19	GMA News	2204	2
49	NTG: Bilang ng mga may edad nang tinatamaan ng tigdas, tumaas<br>https://www.youtube.com/watch?v=ySf-zP1tZVI	20.02.19	GMA News	1113	1
50	NTVL: DOH: Bilang ng measles cases, umakyat na sa halos 7,000<br>https://www.youtube.com/watch?v=FgxCteaj-jY	16.02.19	GMA News	436	0
51	BP: Vaccination program kontra-tigdas, inilunsad ng DOH<br>https://www.youtube.com/watch?v=bh1ojhBIrxU	24.04.18	GMA News	361	0
52	24 Oras: Batang naturukan ng dengvaxia vaccine, namatay sa kumplikasyon<br>https://www.youtube.com/watch?v=8-qZ-gHonRw	24.02.18	GMA News	26361	34
53	BT: Nasa 200 bata, binigyan ng oral polio vaccine sa isang medical mission<br>https://www.youtube.com/watch?v=B5Powkec-3k	22.09.19	GMA News	1547	1
54	Ilang rabies vaccine mula China, pinababawi ng FDA; supply ng bakuna sa bansa, apektado<br>https://www.youtube.com/watch?v=ckgoIRmcv9Y	12.09.18	GMA News	1509	5
55	DOH: Supply ng anti-rabies vaccine sa bansa, kulang mula pa noong Enero<br>https://www.youtube.com/watch?v=JYnAHQuxV4E	28.08.18	GMA News	10662	7
56	Aabot sa 2,000 nabakunahan ng pekeng bakuna kontra-rabies, tinawagan na para masuri<br>https://www.youtube.com/watch?v=7zYD2l8OlUA	31.01.19	GMA News	3182	12
57	Babala ng FDA: May naglipanang pekeng anti-rabies vaccine sa merkado<br>https://www.youtube.com/watch?v=3BBAJ20jR5U	31.01.19	GMA News	9962	24
58	24 Oras: DOH, aminadong posibleng may naglipana pang pekeng anti-rabies vaccine<br>https://www.youtube.com/watch?v=1xnOQDleji4	03.02.19	GMA News	2712	4

59	Pamilya ng mga nagkasakit o namatay dahil umano sa dengvaxia vaccine, desididong magkaso<br>https://www.youtube.com/watch?v=vQN8xCt8D1o	09.01.18	GMA News	3314	12
60	Distributor na nagbenta ng pekeng vaccine, inireklamo na ng Medical City<br>https://www.youtube.com/watch?v=6Q8fv6rf6Gs	31.01.19	GMA News	1027	3
61	BT: 7 bakuna, pinapayong maiturok sa mga sanggol para iwas-sakit<br>https://www.youtube.com/watch?v=QgQ39ut-Sx0	07.02.19	GMA News	4203	1
62	Pinoy MD: Bawal nga bang maligo kapag may bulutong?<br>https://www.youtube.com/watch?v=omZDJhXE52E	10.09.18	GMA Public Affairs	200263	220
63	Pinoy MD: Totoo ba na bawal ang electric fan sa batang may tigdas?<br>https://www.youtube.com/watch?v=mSrYalUEaHk	11.06.19	GMA Public Affairs	94852	34
64	Unang Hirit: Usapang Pangkalusugan: Polio Vaccine, hindi dapat pangambahan<br>https://www.youtube.com/watch?v=q-j8mApz3kI	16.10.19	GMA Public Affairs	4950	4
65	Stand for Truth: Kahalagahan ng bakuna<br>https://www.youtube.com/watch?v=oIYvztUWsGE	10.06.19	GMA Public Affairs	2894	2
66	Reel Time: Naniniwala ka ba sa kahalagahan ng bakuna?<br>https://www.youtube.com/watch?v=npuYQVRu5BE	18.02.19	GMA Public Affairs	15977	13
67	Reporter‘s Notebook: Mga magulang ng mga batang naturukan ng Dengvaxia vaccine, naaalarma!<br>https://www.youtube.com/watch?v=R8LmI6R74Gk	25.01.18	GMA Public Affairs	26458	54
68	Brigada: Proseso sa paglabas ng Dengvaxia vaccine sa merkado, tinalakay sa 'Brigada'<br>https://www.youtube.com/watch?v=meKwJxo4mrs	17.01.18	GMA Public Affairs	4968	8
69	Be careful with getting 4 doses of COVID-19 vaccine: neurosurgeon | ANC<br>https://www.youtube.com/watch?v=ZHF6QatFEg4	12.01.22	ABS-CBN News	649548	2230
70	Side effects from Pfizer’s COVID-19 vaccine are 'tolerable’, Pinoy frontliners in US say<br>https://www.youtube.com/watch?v=VgYd3teFUS0	12.01.21	ABS-CBN News	620809	1216
71	Moderna vaccine vs COVID-19 nagpakita ng magandang resulta sa trials | TV Patrol<br>https://www.youtube.com/watch?v=2lTMvhGA6Jo	15.07.20	ABS-CBN News	311798	495
72	Pinoy sa UAE ibinahagi ang pinagdaanan sa COVID-19 vaccine clinical trial | TV Patrol<br>https://www.youtube.com/watch?v=VMH-j_mZGVU	08.09.20	ABS-CBN News	198250	329
73	Parang fiesta; OFW in UAE inoculated with Sinopharm's COVID-19 vaccine | TeleRadyo<br>https://www.youtube.com/watch?v=gicywkLUknM	15.12.20	ABS-CBN News	196012	783
74	ALAMIN: COVID vaccine, anti-rabies shot, di pwedeng pagsabayin | TeleRadyo<br>https://www.youtube.com/watch?v=z7GRC7xRjKg	26.05.21	ABS-CBN News	38291	395
75	DOH ramps up measles, polio immunization campaign ahead of possible outbreak | ANC<br>https://www.youtube.com/watch?v=Z_7Lh-S-V4Y	21.10.20	ABS-CBN News	3450	11
76	Mga bakuna kontra COVID-19 para sa mga edad 5-11, darating sa Enero: Galvez | TV Patrol<br>https://www.youtube.com/watch?v=j3CyHl8HUlY	27.12.21	ABS-CBN News	2745	0
77	Philippines OKs use of 4 vaccine brands as COVID-19 booster shots | ABS-CBN News<br>https://www.youtube.com/watch?v=7s069eAcR2U	16.11.21	ABS-CBN News	92575	202
78	ALAMIN: Hanggang kailan pwedeng magpaturok ng 2nd dose ng COVID vaccine | TeleRadyo	28.04.21	ABS-CBN News	34276	80

	https://www.youtube.com/watch?v=EZiD-RtxbQY				
79	Mga doktor hinihikayat ang publiko na magpabakuna rin ng flu vaccine | TV Patrol https://www.youtube.com/watch?v=BkacxaWukvc	28.04.22	ABS-CBN News	1941	6
80	Philippines finalizing COVID-19 vaccine rules for 5 to 11-year-olds | ABS-CBN News https://www.youtube.com/watch?v=S24_YNE1gKU	20.01.22	ABS-CBN News	84834	223
81	Pagkamatay ng 3 bata na nabakunahan, walang kinalaman sa COVID-19 vaccine | NXT https://www.youtube.com/watch?v=K_9cK4IoVHk	10.12.21	ABS-CBN News	20348	148
82	Expiration date ng vaccine cards inihirit ng DILG | TV Patrol https://www.youtube.com/watch?v=2KM37Dn_nOw	04.04.22	ABS-CBN News	3514	50
83	Mars Pa More: Iba't ibang kilalang bakuna, makatutulong nga ba laban sa COVID-19? | Momergency https://www.youtube.com/watch?v=BbHXa3RkB5I	14.09.20	GMANetwork	365	0
84	Mars Pa More: Is Covid-19 vaccine booster shot safe? | Momergency https://www.youtube.com/watch?v=83NQKbolfgs	29.09.21	GMANetwork	1558	0
85	Vice Ganda wishes all Filipinos will be vaccinated | Tawag ng Tanghalan https://www.youtube.com/watch?v=B9i7AcHulHE	18.12.20	ABS-CBN Entertainment	13186	10
86	Pagkamatay ng mga matandang may sakit matapos maturukan ng Pfizer vaccine, iniimbestigahan | 24 Oras https://www.youtube.com/watch?v=bamS--f4Ev4	16.01.21	GMA News	1251046	2989
87	COVID-19 vaccine myths, debunked! | Need to Know https://www.youtube.com/watch?v=3-GEYaoMkG4	17.04.21	GMA News	608809	2030
88	Sanggol na aksidenteng nabigyan ng COVID vaccine, nilagnat | 24 Oras Weekend https://www.youtube.com/watch?v=rdXKTxfGiXY	08.01.22	GMA News	527715	1563
89	Mga walang vaccine card o di kaya'y 'di pa bakunado, hindi pinalulusot sa checkpoint | BT https://www.youtube.com/watch?v=PMJGb0axQvQ	10.01.22	GMA News	515193	1033
90	79 kaso ng pamumuo ng dugo matapos bigyan ng AstraZeneca vaccine, naiulat sa U.K.; 19 patay | UB https://www.youtube.com/watch?v=gl8frDEMTfg	08.04.21	GMA News	489462	748
91	Pagbabakuna kontra measles, rubella, tetanus at diptheria, ginawang bahay-bahay... | 24 Oras https://www.youtube.com/watch?v=I8Gq6p_udl0	12.11.21	GMA News	3976	0
92	11-anyos na bata, naturukan umano ng bakuna na hindi angkop sa kanyang edad | UB https://www.youtube.com/watch?v=o-reiLb5yIA	10.02.22	GMA News	26508	59
93	Philippine vaccine experts' decision on boosters may be out next week —official | 24 Oras https://www.youtube.com/watch?v=5cG2bn_3a1I	03.09.21	GMA News	20421	41
94	Mahigit 80 na kinalaman sa pekeng bakuna kontra-COVID-19, huli sa China | 24 Oras https://www.youtube.com/watch?v=DyrqhbiYCRA	03.02.21	GMA News	13355	51
95	Pinoy MD: Mga hindi dapat gawin pagkatapos mabakunahan https://www.youtube.com/watch?v=5sbYg1J5eoE	07.09.21	GMA Public Affairs	80383	62

96	COVID-19 vaccine, may epekto ba sa buntis? | Pinoy MD<br>https://www.youtube.com/watch?v=gOG2DUBQdVw	04.06.22	GMA Public Affairs	1977	0
97	Unang Hirit: Pinay na naturukan ng Moderna COVID-19 vaccine, nakaranas ng side effects<br>https://www.youtube.com/watch?v=JBZOHqvQ2RY	15.02.21	GMA Public Affairs	406442	702
98	Duterte: Philippines' first COVID-19 vaccine might come from China | ABS-CBN News<br>https://www.youtube.com/watch?v=RBOtpAfaeo4	31.07.20	ABS-CBN News	457772	1841
99	Philippines; slow COVID-19 vaccine procurement;embarrassing; disgusting: Lacson | TeleRadyo<br>https://www.youtube.com/watch?v=FLJSTUb54Mc	05.01.21	ABS-CBN News	424934	937
100	British-Pinay nurse na unang nagturok ng COVID-19 vaccine, pinarangalan sa UK | TV Patrol<br>https://www.youtube.com/watch?v=UIzzRssYAvA	13.07.22	ABS-CBN News	253845	183
101	Love Together, Hope Together: Bakuna together | GMA Christmas Station ID 2021<br>https://www.youtube.com/watch?v=g2yo-s9f03w	24.11.21	GMANetwork	9037	10
102	Vice teases Vhong about the Vaccine | It's Showtime<br>https://www.youtube.com/watch?v=TlrmYXiKEJc	26.02.21	ABS-CBN Entertainment	99180	36
103	Vice Ganda reveals why he got vaccinated | It's Showtime<br>https://www.youtube.com/watch?v=WulMd2Y9c4U	01.07.21	ABS-CBN Entertainment	216438	134
104	Babala ng DOH sa mga nagpa-COVID vaccine booster shot: Hindi accountable ang... | 24 Oras News Alert<br>https://www.youtube.com/watch?v=AmTIE7uZ8do	12.11.21	GMA News	641768	1032
105	24 Oras: Negosyanteng nagpaturok na ng COVID-19 vaccine mula China, may nakasabay na mga pulitiko<br>https://www.youtube.com/watch?v=HNZdJCBQ2MY	17.12.20	GMA News	576892	1343
106	Flying Vaccine Deliver, paano sinimulan? | Unang Hirit<br>https://www.youtube.com/watch?v=98HxWBBcub8	25.04.22	GMA Public Affairs	4147	1
107	Ano ang side effects ng Pfizer at Moderna COVID-19 vaccine? | NXT<br>https://www.youtube.com/watch?v=aVWqdcbPSQ4	11.05.21	ABS-CBN News	2237043	1913
108	Bakit nagkakaroon ng side effects ang mga bakuna sa COVID-19? | NXT<br>https://www.youtube.com/watch?v=N4HlqVogHC0	13.04.21	ABS-CBN News	1260181	1622

Note: View- and comment-counts as per August 2, 2022.
