# Supplementary material for: Medical knowledge, political tension, and social relevance: a content and framing analysis of vaccine-related TV broadcasts in the Philippines
Source: BMJ Public Health. 2025 Jul 25;3(2):e002133. doi: 10.1136/bmjph-2024-002133 (PMC12306340; doi:10.1136/bmjph-2024-002133)
Supplement: online supplemental file 4 [file bmjph-3-2-s004.pdf]

Medical Knowledge, Political Tension, Social Relevance: A content and framing analysis of Vaccine-Related TV Broadcasts in the Philippines.

Supplemental file 4. Videos selected for framing analysis.

Table S3: Videos selected for framing analysis

	Name	Upload Date	Channel	Views	Comments
1	DOH: Ligtas ang libreng bakuna<br>https://www.youtube.com/watch?v=ZFISVTO3FO0	19.08.2015	ABS-CBN News	8985	0
2	TV Patrol: Anti-dengue vaccine, ligtas ba?<br>https://www.youtube.com/watch?v=rYWfhZ3HbXg	31.03.2016	ABS-CBN News	9919	11
3	BT: Pagkamatay ng 2 sanggol dahil umano sa bakuna, iniimbestigahan ng DOH<br>https://www.youtube.com/watch?v=1lk9U33NDzI	16.11.2017	GMA News	33862	24
4	Pinoy MD: Sakit na pneumonia, paano nga ba maiiwasan?<br>https://www.youtube.com/watch?v=RfU7wGtNvZY	25.11.2017	GMA Public Affairs	461221	109
5	Philippines suspends dengue vaccine program after Sanofi warns of risks<br>https://www.youtube.com/watch?v=n5qoqKZNvGM	01.12.2017	ABS-CBN News	22231	49
6	Pamilya ng mga nagkasakit o namatay dahil umano sa dengvaxia vaccine, desididong magkaso<br>https://www.youtube.com/watch?v=vQN8xCt8D1o	09.01.2018	GMA News	3314	12
7	Salamat Dok: Importance of measles vaccines<br>https://www.youtube.com/watch?v=5HZ4Ny2c1LM	09.12.2018	ABS-CBN News	10089	3
8	TV Patrol: PAO itinangging sila ang dahilan ng ‘vaccine scare’<br>https://www.youtube.com/watch?v=e6HTw-ERTsY	18.12.2018	ABS-CBN News	822	3
9	Pati mga fast food chain, nilagyan na ng vaccination site<br>https://www.youtube.com/watch?v=nu9rgVWopLM	12.02.2019	GMA News	2204	2
10	Bandila: DOH - Kumpletuhin ang bakuna ng mga bata<br>https://www.youtube.com/watch?v=L6NdJSGD13k	01.03.2019	ABS-CBN News	8918	5
11	Vice Ganda wishes all Filipinos will be vaccinated | Tawag ng Tanghalan<br>https://www.youtube.com/watch?v=B9i7AcHulHE	18.12.2020	ABS-CBN Entertainment	13186	10
12	Vice teases Vhong about the Vaccine | It's Showtime<br>https://www.youtube.com/watch?v=TlrmYXiKEJc	26.02.2021	ABS-CBN Entertainment	99180	36
13	Ano ang side effects ng Pfizer at Moderna COVID-19 vaccine? | NXT<br>https://www.youtube.com/watch?v=aVWqdcbPSQ4	11.05.2021	ABS-CBN News	2237043	1913
14	Vice Ganda reveals why he got vaccinated | It's Showtime<br>https://www.youtube.com/watch?v=WulMd2Y9c4U	01.07.2021	ABS-CBN Entertainment	216438	134
15	Mars Pa More: Is Covid-19 vaccine booster shot safe? | Momergency<br>https://www.youtube.com/watch?v=83NQKbolfgs	29.09.2021	GMA Network	1558	0
16	Love Together, Hope Together: Bakuna together | GMA Christmas Station ID 2021<br>https://www.youtube.com/watch?v=g2yo-s9f03w	24.11.2021	GMA Network	9037	10

Note: View- and comment-counts as per August 2, 2022.
